# The effect of the volemic and cardiac status on brain oxygenation in patients with subarachnoid hemorrhage: a bi-center cohort study

**DOI:** 10.1186/s13613-021-00960-z

**Published:** 2021-12-16

**Authors:** Verena Rass, Elisa Gouvea Bogossian, Bogdan-Andrei Ianosi, Lorenzo Peluso, Mario Kofler, Anna Lindner, Alois J. Schiefecker, Lauma Putnina, Max Gaasch, Werner O. Hackl, Ronny Beer, Bettina Pfausler, Fabio Silvio Taccone, Raimund Helbok

**Affiliations:** 1 Neurological Intensive Care Unit, Department of Neurology, Medical University of Innsbruck, Anichstrasse 35, 6020 Innsbruck, Austria; 2 Department of Intensive Care, Erasme Hospital, Université Libre de Bruxelles, Route de Lennik, 808, 1070 Brussels, Belgium; 3 Institute of Medical Informatics, UMIT: University for Health Sciences, Medical Informatics and Technology, Eduard Wallnoefer-Zentrum 1, 6060 Hall, Austria

**Keywords:** Subarachnoid hemorrhage, Fluid management, PiCCO, Critical care, Multimodal neuromonitoring, Brain oxygenation

## Abstract

**Background:**

Fluid management in patients after subarachnoid hemorrhage (SAH) aims at the optimization of cerebral blood flow and brain oxygenation. In this study, we investigated the effects of hemodynamic management on brain oxygenation by integrating advanced hemodynamic and invasive neuromonitoring.

**Methods:**

This observational cohort bi-center study included data of consecutive poor-grade SAH patients who underwent pulse contour cardiac output (PiCCO) monitoring and invasive neuromonitoring. Fluid management was guided by the transpulmonary thermodilution system and aimed at euvolemia (cardiac index, CI ≥ 3.0 L/min/m^2^; global end-diastolic index, GEDI 680–800 mL/m^2^; stroke volume variation, SVV < 10%). Patients were managed using a brain tissue oxygenation (P_bt_O_2_) targeted protocol to prevent brain tissue hypoxia (BTH, P_bt_O_2_ < 20 mmHg). To assess the association between CI and P_bt_O_2_ and the effect of fluid challenges on CI and P_bt_O_2_, we used generalized estimating equations to account for repeated measurements.

**Results:**

Among a total of 60 included patients (median age 56 [IQRs 47–65] years), BTH occurred in 23% of  the monitoring time during the first 10 days since admission. Overall, mean CI was within normal ranges (ranging from 3.1 ± 1.3 on day 0 to 4.1 ± 1.1 L/min/m^2^ on day 4). Higher CI levels were associated with higher P_bt_O_2_ levels (Wald = 14.2; *p* < 0.001). Neither daily fluid input nor fluid balance was associated with absolute P_bt_O_2_ levels (*p* = 0.94 and *p* = 0.85, respectively) or the occurrence of BTH (*p* = 0.68 and *p* = 0.71, respectively). P_bt_O_2_ levels were not significantly different in preload dependent patients compared to episodes of euvolemia. P_bt_O_2_ increased as a response to fluid boluses only if BTH was present at baseline (from 13 ± 6 to 16 ± 11 mmHg, OR = 13.3 [95% CI 2.6–67.4], *p* = 0.002), but not when all boluses were considered (*p* = 0.154).

**Conclusions:**

In this study a moderate association between increased cardiac output and brain oxygenation was observed. Fluid challenges may improve P_bt_O_2_ only in the presence of baseline BTH. Individualized hemodynamic management requires advanced cardiac and brain monitoring in critically ill SAH patients.

**Supplementary Information:**

The online version contains supplementary material available at 10.1186/s13613-021-00960-z.

## Introduction

Optimal fluid management is crucial after subarachnoid hemorrhage (SAH) to maintain adequate cerebral blood flow (CBF), particularly in the setting of impaired autoregulatory capacity, and aims at the prevention of secondary brain injuries [1]. Evidence suggests that hypoperfusion and hypotension are associated with an increased risk of brain damage, requiring prompt fluid resuscitation, especially in the early period after the initial bleeding [2]. In contrast, fluid overload can lead to complications, such as pulmonary or cerebral edema, and has been associated with poor functional outcome [3, 4]; as such, international guidelines advocate the achievement of “euvolemia” in SAH patients [1].

 However, monitoring of the fluid status and intravascular volume can be challenging in this setting. Traditional methods, such as the assessment of fluid balance and/or monitoring of vital signs [1], the use of echocardiography [5], or passive leg raising maneuvers [6], may not be sufficient in poor-grade SAH patients with hemodynamic impairment, including myocardial stunning [7]. The semi-invasive transpulmonary thermodilution system has been developed to achieve a goal-directed hemodynamic support and optimal fluid management. It allows for accurate prediction of fluid responsiveness at the bedside, by measuring the cardiac output and the volume status simultaneously [8]. Several studies showed its feasibility in SAH patients, and a goal-directed therapy based on this hemodynamic monitoring may potentially decrease the occurrence of delayed cerebral ischemia (DCI) and improve the management of hemodynamically instable SAH patients [9–11].

Individualized therapy to specifically reduce secondary brain injuries may be accomplished by the use of invasive multimodal neuromonitoring in poor-grade SAH patients [12, 13]. Brain tissue oxygenation (P_bt_O_2_) is an important physiologic target and reflects the equilibrium between cerebral perfusion, oxygen delivery, oxygen consumption, and tissue diffusion into the brain parenchyma [14]. A pilot study, including 10 SAH patients, reported that fluid challenges resulted in an improved cardiac output and subsequently increased P_bt_O_2_ levels, particularly in those who were “fluid responders” [15]. Another study showed that hypervolemia, as part of the previously recommended hypertension, hypervolemia and hemodilution (triple-H) therapy, did not result into improved P_bt_O_2_ levels [16]. These studies underline that hemodynamic interventions should be individualized on brain monitoring, such as brain oxygenation, to expect beneficial cerebral effects in SAH patients.

The aim of the current study was therefore to investigate the effect of fluid management and the cardiac function on brain oxygenation, by integrating advanced hemodynamic and invasive neuromonitoring tools. We hypothesized that a higher cardiac index (CI) would be associated with a better brain tissue oxygenation and that fluid challenges would result into a significant increase in brain tissue oxygenation.

## Methods

### Study design, setting, and participants

This study was designed according to the STROBE statement on observational cohort studies. We prospectively collected data of patients admitted to the ICU of two tertiary care centers (Medical University of Innsbruck, Austria; Erasme Hospital, Brussels, Belgium) between 2010 and 2019 diagnosed of non-traumatic subarachnoid hemorrhage. Inclusion criteria were as follows: (1) age ≥ 18 years; (2) neuromonitoring of intracranial pressure (ICP) and P_bt_O_2_ as part of clinical routine; and (3) concurrent advanced hemodynamic monitoring with a transpulmonary thermodilution system (Pulse Contour Cardiac Output, PiCCO; Pulsion Medical Systems SE, Munich, Germany). This study was approved by the local ethics committees (Medical University Innsbruck, AN3898 285/4.8, AM4091-292/4.6; Erasme Hospital Brussels, P2019/649). All patients in Innsbruck (IBK) gave informed consent according to local regulations and it was waived in Brussels (BRU). The conduct of this study conformed to the Declaration of Helsinki.

### Clinical management and grading

Patients were clinically graded with use of the Hunt&Hess (H&H) grade on admission; poor grade was defined as scores of 4 or 5. The management conformed to international guidelines [17, 18], except for nimodipine which was administered intravenously in poor-grade patients in Innsbruck. Aneurysms were secured with means of neurosurgical clipping or endovascular coiling, after multi-disciplinary discussion in both centers. All patients were regularly followed for large-vessel vasospasm with daily transcranial color-coded duplex sonography (TCCS). Vasospasm was diagnosed in the setting of mean velocities > 120 cm/s in the anterior and/or middle cerebral artery or daily changes of > 50 cm/s. Delayed cerebral ischemia was defined as the occurrence of a new focal neurologic deficit, a decrease of at least two points on the Glasgow Coma Scale, or a new infarct on the computed tomography (CT) or magnetic resonance imaging scan not attributable to other causes [19]. All patients were mechanically ventilated and received continuous drips of sufentanil, midazolam, propofol and/or ketamine, according to local practices.

### Hemodynamic monitoring and fluid management

The use of advanced hemodynamic monitoring was based on (1) hemodynamic instability or (2) the presence of stunned myocardium on echocardiography. Continuous values of the CI and stroke volume variation (SVV) were recorded. Transpulmonary thermodilution was performed three times per day to calibrate for the assessment of cardiac volumes, extravascular lung water, using a 15 mL of cold (< 8 °C) 0.9% saline bolus injected into the superior vena cava. Based on the thermodilution curves, estimations of the CI (normal range: 3.0–5.0 L/min/m^2^), global end-diastolic volume index (GEDI; 680–800 mL/m^2^), extravascular lung water index (ELWI; < 7 mL/kg), and systemic vascular resistance index (SVRI; 1700–2400 dyn·s m^2^/cm^5^) were obtained [8]. SVV was only interpreted in the setting of a fully controlled ventilation mode (73% of the monitoring time). In the setting of low GEDI (< 680 mL/m^2^) or high SVV (> 10%), patients were considered preload dependent.

Fluid management aimed at euvolemia and was directed by the PiCCO method by targeting normal CI, GEDI, SVV levels, and avoidance of fluid overload indicated by a high ELWI, signs of pulmonary edema on chest radiography, and blood gas analyses (i.e., low Horovitz index), and signs of peripheral edema in the absence of hypoalbuminemia and capillary leakage syndrome [20]. Euvolemia was maintained by volume replacement with isotonic crystalloids (IBK: Elomel Fresenius Kabi Austria, Graz, Austria, 302 mOsm/L 2014–2016 and Sodium-chloride 0.9%, 308 mOsm/L; Braun, Melsungen, Germany until 2014; BRU: Plasmalyte, Baxter Healthcare Ltd or Sodium-chloride 0.9%, 308 mOsm/L Baxter Healthcare Ltd) and fluid resuscitation was achieved with colloids or crystalloids (IBK: Gelofusin 274 mOsm/L; Braun, Melsungen, Germany; BRU: Plasmalyte, Baxter Healthcare Ltd). Diuretics (mainly furosemide) were used if necessary in the setting of fluid overload or decreased urine output. For hemodynamic augmentation, vasopressors (mainly noradrenaline) or fluids were used, as decided by the treating physician. Neurogenic stunned myocardium was diagnosed based on electrocardiographic and echocardiographic abnormalities associated with elevated troponin levels and the requirement for continuous inotropic drugs (i.e., milrinone or dobutamine) to improve impaired left ventricular function (if CI < 3.0 L/min/m^2^) [21]. In the presence of suspected hypocortisolism and high dependence on vasopressors, hydrocortisone (IBK: 8 mg/h as continuous drip, BRU: 50 mg q6h) was given.

### Invasive multimodal neuromonitoring

The indications for invasive multimodal monitoring (ICP and P_bt_O_2_) were as follows: (1) poor-grade SAH; (2) anticipated prolonged mechanical ventilation; and (3) clinical or radiological signs suggestive of raised intracranial pressure. Neuromonitoring probes were placed into the hemisphere at greatest risk of secondary brain injury either through a frontal burr hole using a triple-lumen bolt or tunneled and placed in the white matter. Probe location was confirmed by brain CT scans obtained within 24 h from implantation. Brain oxygenation catheters (Licox^®^ CC1.SB probes, Integra LifeSciences, Ratingen, Germany) and intraparenchymal ICP probes (Neurovent-P-temp, Raumedic^®^, Helmbrechts, Germany) were used. Cerebral perfusion pressure was calculated by subtracting ICP from mean arterial pressure (MAP), measured at the level of the Monroe foramen (IBK) or at the level of the heart (BRU) which accounts for approximately 15 mmHg difference, with higher CPP levels when measured at the level of the heart. In clinical practice, brain tissue hypoxia (BTH) was treated if P_bt_O_2_ < 20 mmHg lasted for > 5 min according to local protocols (Additional file 1: Figs. S1, S2). Treatment options included increases of CPP ≥ 70 mmHg (Monroe level; IBK) or 90–100 mmHg (heart level; BRU) with vasopressors or fluid administration as indicated, targeting normocapnia (IBK: PaCO_2_ > 35 mmHg; BRU: PaCO_2_ ≥ 40 mmHg; with consideration of ICP levels), optimization of oxygenation (IBK: PaO_2_ ≥ 80 mmHg, BRU: PaO_2_ 100–120 mmHg), RBC transfusions in anemic patients (IBK goal Hb ≥ 8 g/dL, BRU goal Hb ≥ 9 g/dL) and titration of analgesia, and sedation. For the current study, we used the definition of BTH when median hourly levels of P_bt_O_2_ were below 20 mmHg. Multimodal neuromonitoring was performed in the most vulnerable phase when patients were at risk of BTH and/or ICP crises in accordance with the manufacturer’s recommendations.

### Data collection

We prospectively collected demographics, hospital complications, and outcomes. Functional outcome was evaluated three months after the ictus with the use of the modified Rankin Scale Score (mRS), and poor functional outcome was defined as mRS > 2. Continuous variables, including PiCCO parameters (CI, SVV), neuromonitoring parameters (P_bt_O_2_, ICP, CPP), heart rate (HR), and MAP, were saved in a granularity of 3 min (IBK) or 15 min (BRU) using an electronic patient data management system (IBK: CentricityTM Critical Care 8.1 SP7; GE Healthcare Information Technology, Dornstadt German; BRU: CareSuite 8.2.7.758 ICU; PICIS, N. Harris Computer Corporation Inc.) and calculated as hourly medians for the purposes of this study. In a subset of 18 patients (IBK) with available high-resolution data, we estimated the autoregulation state by using the Pressure reactivity index (PRx), calculated as a moving Pearson correlation between 30 consecutive 10-s averages of ICP and MAP, resulting in a moving 5-min time window, incremented in 10-s steps. [22].

Multimodal neuromonitoring parameters were checked for plausibly and artifacts were deleted manually. We discarded P_bt_O_2_ levels recorded within the initial stabilization time (at least 2 h of insertion). The following intermittent variables were derived from our patient data management systems, whenever available: daily sums of fluid input and balance, daily sums of vasopressor doses, the timing and volume of crystalloids and colloids, and PiCCO parameters (GEDI, ELWI, SVRI).

### Study outcome measures

The primary study outcome measure was the effectiveness of fluid challenges on brain oxygenation. Fluid challenges were defined by the administration of 500–1000 mL colloid or crystalloid solution over 30–60 min. Further, we evaluated whether an association between the cardiac performance as assessed with CI and brain oxygenation exists.

### Statistical analyses

We decided to analyze the first 10 days after bleeding to avoid a selection bias toward the most severe patients. Data availability was limited thereafter. The first day (first 24 h after admission) was denoted as day 0. We predefined time periods as follows: days 0–2 (representing the time of early brain injury), days 3–4, and days 5–9. Based on the observation that BTH most commonly occurred within the first 24 h, we performed separate analyses for days 1–9 to test whether the effects hold true after the initial stabilization time. The prevalence rates of BTH and CI ranges are given as percentage of the overall monitored time or as daily percentage, as indicated. Categorical variables are given as counts and percentage, continuous variables as median and interquartile range or mean and standard deviation. Based on variable distribution (tested by the Kolmogorov–Smirnov test and Shapiro–Wilk test), parametric and non-parametric procedures, including the *t*-test and Mann–Whitney *U* test, were used to assess differences between patients from the two centers. Categorical variables were analyzed using the Chi-squared and Fishers exact test. Univariate and/or multivariable generalized estimating equation (GEE) models were used for all analyses of repeated measurements within one subject with an autoregressive process of the first order [23]. Covariates for multivariable models were preselected based on their clinical significance and specified accordingly. Changes of CI (∆CI) and P_bt_O_2_ (∆P_bt_O_2_) were calculated as the difference between two consecutive hourly median values.

To study the effect of fluid challenges on CI and P_bt_O_2_, we also used GEE models with three time points: 1 h before administration (= baseline), during (one hour) administration, and 1 h after administration and used the median hourly values of selected variables. Separate analyses were done for CI responders (CI increase > 10% from baseline) [15] vs. CI non-responders, for colloids vs. crystalloids and for the presence of BTH or normal brain tissue oxygenation at baseline. A *p*-value < 0.05 was considered statistically significant. All analyses were performed with SPSS (IBM SPSS Statistics, Version 24.0 Armonk, NY, USA).

## Results

### Study population

Out of 167 (IBK: 81, BRU: 86) patients monitored with multimodal neuromonitoring, a total of 60 patients (IBK: 39, BRU: 21) met the inclusion criteria and were eventually included in the final analysis. Most patients were admitted with a poor H&H grade of 4–5 (62%) or deteriorated within 24 h (38%) of admission. The median age was 56 (47–65) years and 37 (62%) of patients were female; 73% of them had poor functional outcome at the 3-month follow-up. Detailed information on patients’ demographics, hospital complications, and outcomes are given in Table 1; most characteristics were not significantly different across the two centers (Additional file 1: Table S1).

**Table 1:** Baseline characteristics, complications, and outcomes

Clinical characteristics		N = 60
Age in years		56 (47–65)
Female sex		37 (62)
Chronic heart failure		0 (0)
Previous myocardial infarction		3 (5)
Pre-existing hypertension		22 (37)
Smoking history		24 (40)
Admission H&H grade	1	3 (5)
	2	6 (10)
	3	14 (23)
	4	6 (10)
	5	31 (52)
GCS at ICU admission		3 (3–10)
LOC at ictus		36 (60)
**Admission radiological characteristics**		
Modified Fisher Scale	1	2 (3)
	2	2 (3)
	3	13 (22)
	4	43 (72)
ICH present on admission CT scan		30 (50)
Hydrocephalus requiring EVD placement		42 (70)
Global cerebral edema		24 (40)
**Aneurysm treatment**		
Endovascular coiling		31 (52)
Neurosurgical clipping		27 (45)
Non-aneurysmal SAH^a^		2 (3)
**Complications and treatment**		
Neurogenic myocardial stunning		23 (38)
Pneumonia		42 (70)
Ventriculitis		7 (12)
Vasospasm		48 (80)
Delayed cerebral ischemia		25 (42)
Noradrenaline, daily dose in mg^b^		11 (5–25)
Dobutamine, daily dose in mg^b^		33 (0–258)
Phenylephrine, daily dose in mg^b^		0 (0–3)
**Outcome characteristics**		
Length of ICU stay in days		28 (18–39)
In-hospital mortality		14 (23)
3-month mRS	0	2 (3)
	1	8 (13)
	2	6 (10)
	3	10 (17)
	4	9 (15)
	5	10 (17)
	6	15 (25)

Data are given in median (IQR) and counts (%)

*SAH* subarachnoid hemorrhage, *H&H* Hunt and Hess, *LOC* loss of consciousness, *ICH* intracerebral hemorrhage, *EVD* external ventricular drain, *mRS* modified Rankin Scale

^a^Aneurysm negative in repeated cerebral angiogram 2–3 weeks apart

^b^Within the first 10 days

### *P*_*bt*_*O*_*2*_* and CI over time*

**Fig. 1:**
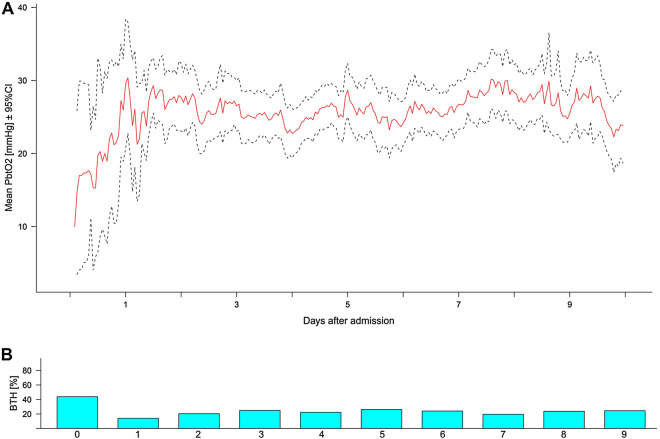
**A** Mean P_bt_O_2_ values and (**B**) daily percentage of brain tissue hypoxia (P_bt_O_2_ < 20 mmHg) over the study time of 10 days

We analyzed a total of 353 days representing a median of 6 (4–8) days per patient. Brain tissue hypoxia occurred in 23% of the monitored time, with the highest prevalence observed on the first day of monitoring (44%; Fig. 1). Almost every patient (87%) developed one episode of BTH during the monitoring time, and the median time each patient spent with BTH was 20 (4–44) % over the study time. The prevalence of BTH (*p* = 0.786) and absolute P_bt_O_2_ (*p* = 0.611) were comparable across the study centers. Lower P_bt_O_2_ levels were associated with poor functional 3-month outcome after correction for age and admission H&H score (adjOR = 0.99 [95% CI = 0.98–0.99], *p* = 0.011).

**Fig. 2:**
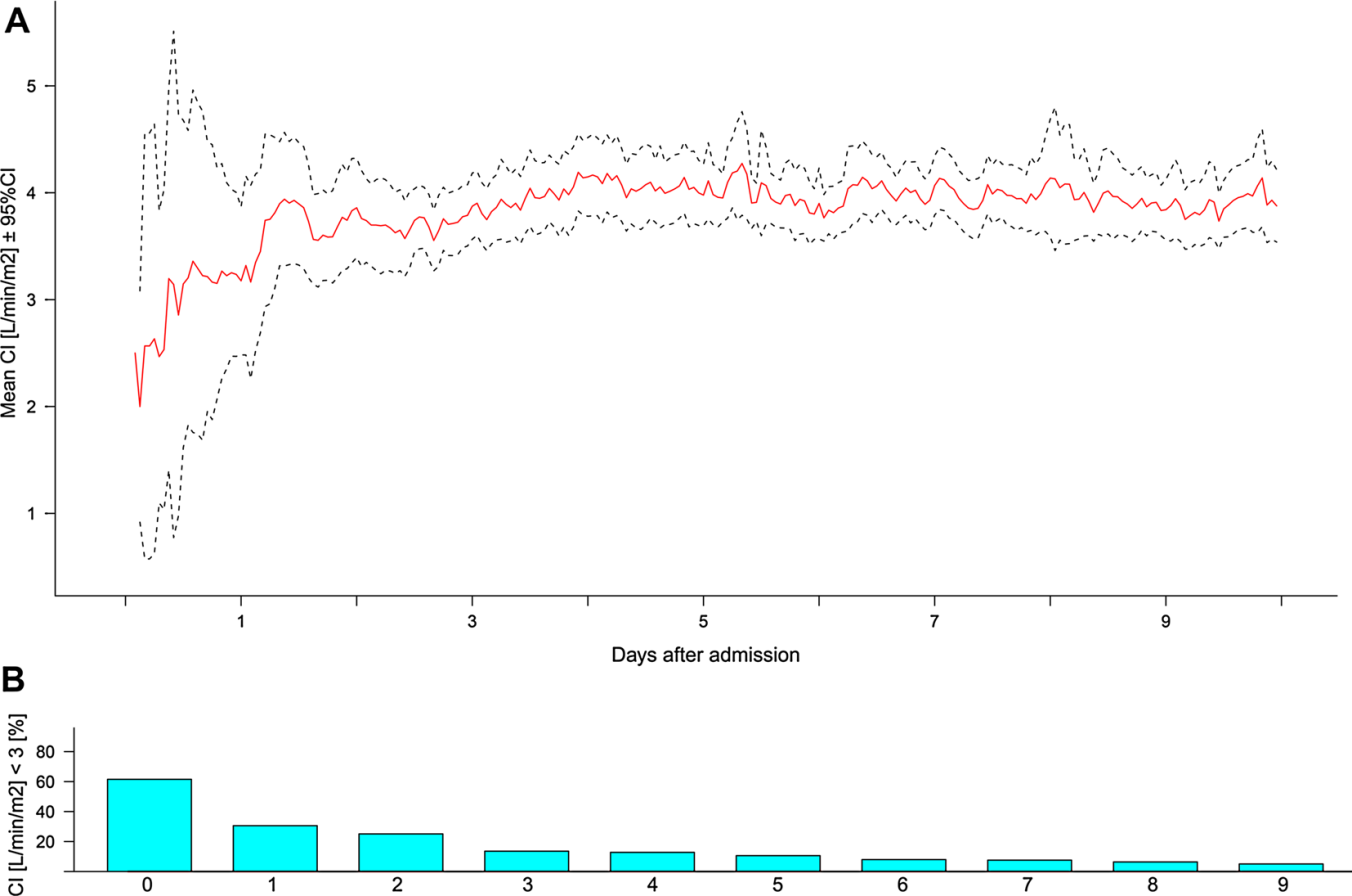
**A** Mean CI values and (**B**) daily percentage of CI < 3 L/min/m^2^ over the study time of 10 days

CI < 3.0 L/min/m^2^ was observed in 14%, CI between 3.0 and 5.0 L/min/m^2^ in 75%, and CI > 5.0 L/min/m^2^ in 11% of the monitored time. This was reflected in mean daily CI levels being constantly within targeted levels ≥ 3.0 L/min/m^2^, ranging from 3.1 ± 1.3 on day 0 to 4.1 ± 1.1 L/min/m^2^ on day 4 (Fig. 2). Co-occurrence of CI values < 3.0 L/min/m^2^ and BTH only occurred in a minority of episodes (3%) of the monitored time.

### *The association between CI and P*_*bt*_*O*_*2*_* or BTH*

**Fig. 3:**
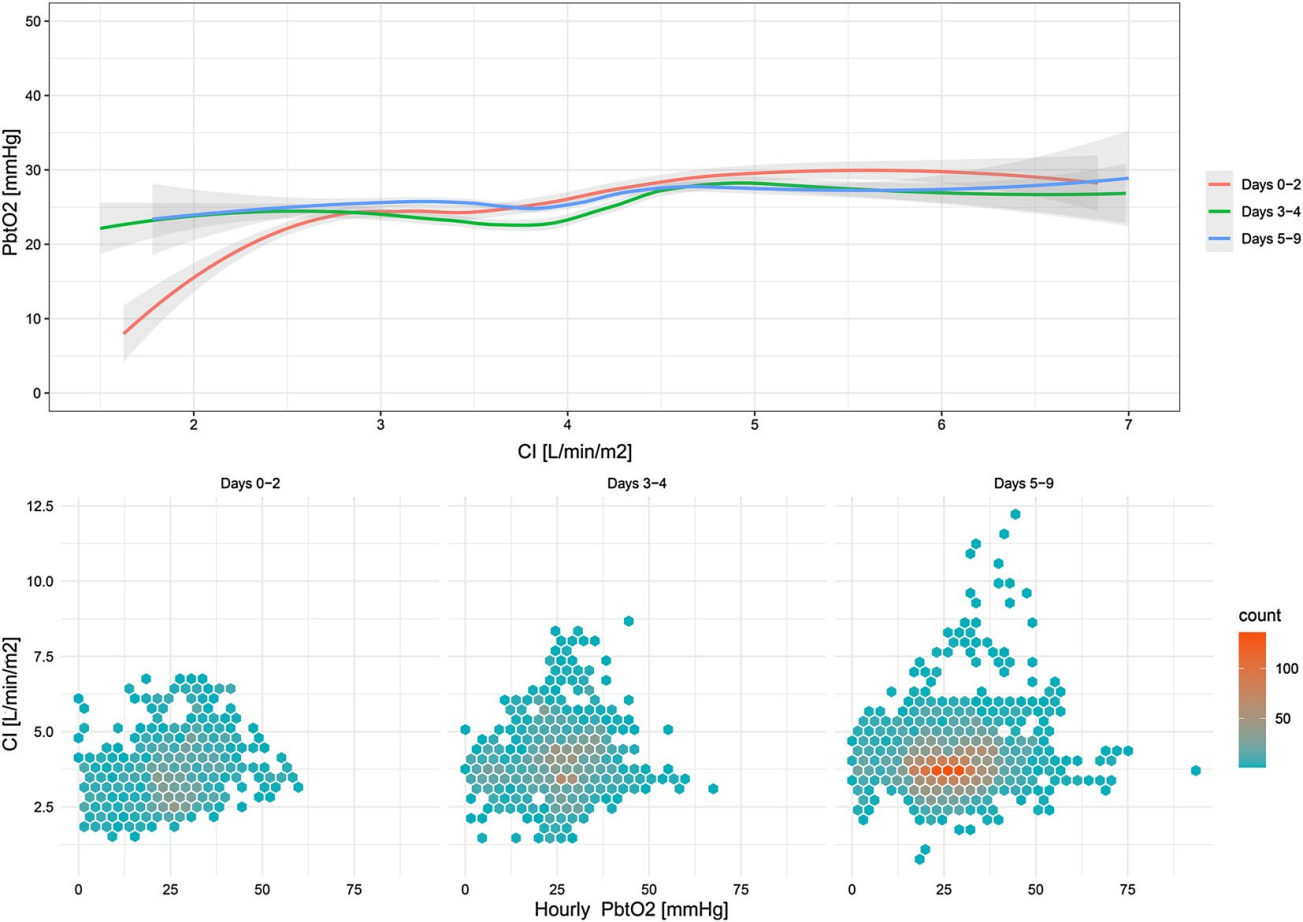
A positive association between CI and P_bt_O_2_ levels was evident across all time phases. The line represents the local polynomial regression and the gray shades represent 95% CI

Higher CI values were associated with higher P_bt_O_2_ levels (Wald = 14.2; *p* < 0.001) throughout the whole study time (days 0–2 *p* = 0.027; days 3–4 *p* = 0.018; days 5–9 *p* = 0.030; days 1–9 *p* < 0.001; Fig. 3). The association between CI and P_bt_O_2_ remained significant even after adjustment for the H&H grade, age, MAP, ICP, PaO_2_, and interaction of CPP * CI (Wald = 6.8 *p* = 0.009; Additional file 1: Table S2). The same results were obtained in a subanalysis of 18 patients with autoregulation recordings: higher CI values were linked to higher P_bt_O_2_ levels after adjustment for PRx (Wald = 10.9, *p* = 0.001).

Mean CI values were higher during normal brain tissue oxygenation measurements when compared to episodes of BTH (4.0 ± 1.0 vs. 3.8 ± 0.8 L/min/m^2^; *p* = 0.042). Accordingly, patients with higher CI levels had decreased odds of BTH (per 0.5 CI increase; OR = 0.88, [95% CI 0.78–0.995], *p* = 0.042). The association was particularly significant within days 3–4 (OR = 0.82, [95% CI 0.68–0.98], *p* = 0.026), but not within days 0–2 (*p* = 0.20) or days 5–9 (*p* = 0.21). A higher ∆CI was associated with a higher ∆P_bt_O_2_ (Wald = 10.5, *p* < 0.001; days 1–9: Wald = 9.7, *p* = 0.002). We found a significant effect of the interaction between the presence of BTH and CI (*p* < 0.001); however, the association between a higher ∆CI and a higher ∆P_bt_O_2_ held true when including the interaction in the model (Wald = 10.5, *p* = 0.001). Moreover, adjusting for PRx did not change the results in 18 patients (Wald = 8.8, *p* = 0.003). CI was abnormal (< 3.0 L/min/m^2^) in the presence of normal P_bt_O_2_ levels ≥ 20 mmHg in 10% of episodes.

### *Neurogenic stunned myocardium and P*_*bt*_*O*_*2*_

**Fig. 4:**
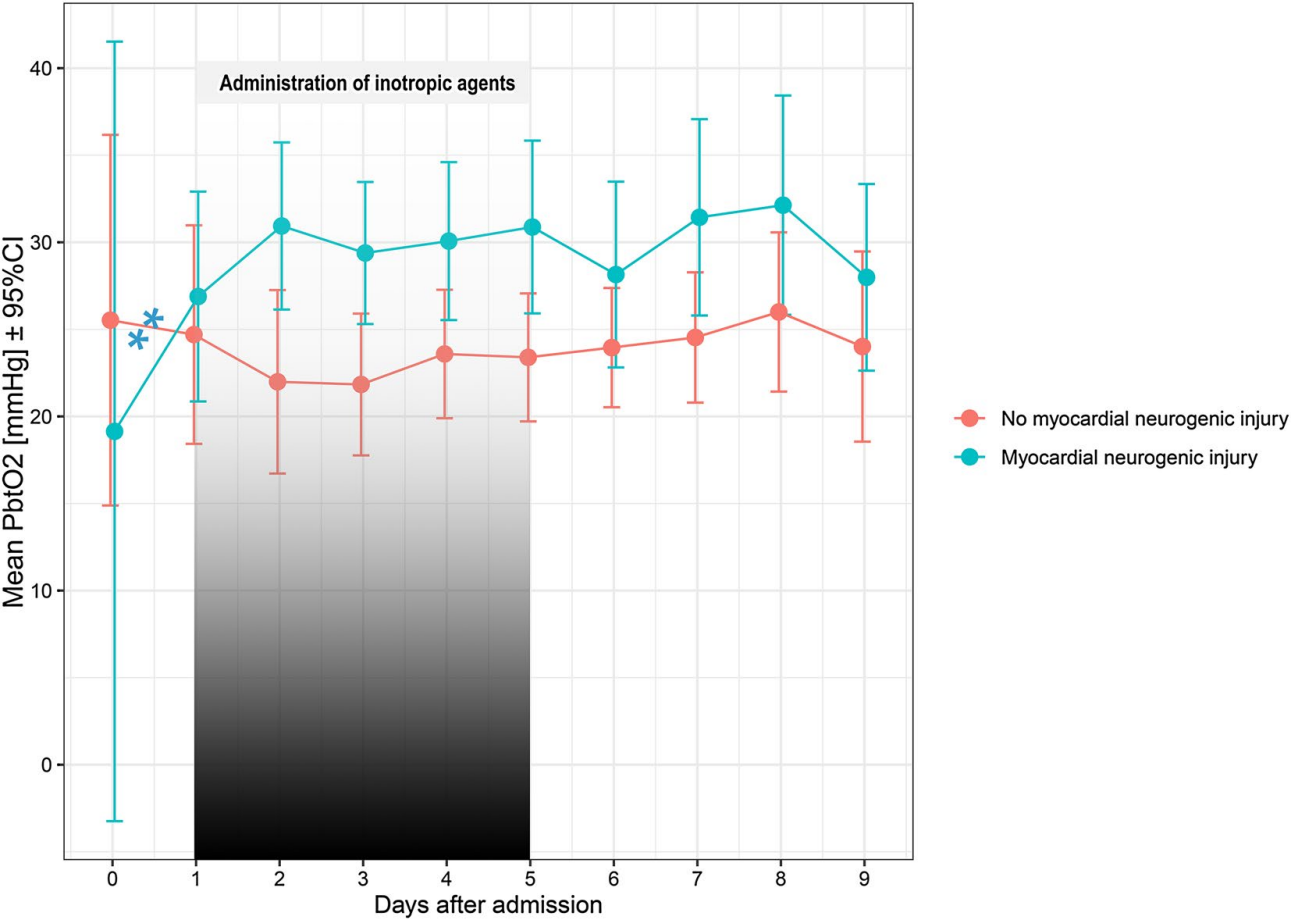
Patients with stunned myocardium had higher P_bt_O_2_ levels as compared to those without stunned myocardium (*p* = 0.005). P_bt_O_2_ levels (*p* = 0.003) rose from day 0 to day 1 in patients with stunned myocardium. The median administration of inotropic agents ranged from days 1 (1–3) to 5 (4–8) within the monitoring time

Twenty-three (38%) patients developed neurogenic stunned myocardium after the bleeding and 21/23 (91%) were treated with inotropic drugs. In these patients, P_bt_O_2_ levels were higher as compared to those without stunned myocardium (29 ± 10 vs. 24 ± 11 mmHg, *p* = 0.005, Fig. 4) over the study period (days 1–9: 30 ± 10 vs. 24 ± 10 mmHg; *p* = 0.004). When adjusting the model for CI, MAP, ICP, PaO_2_, H&H grade, and age, the results remained similar (*p* = 0.003). Accordingly, CI (*p* = 0.83) and MAP levels (*p* = 0.20) were similar across these groups. P_bt_O_2_ levels (per day, OR = 6.1 [95% CI = 1.9–20.2], *p* = 0.003) and CI levels (per day, OR = 1.3 [95% CI = 1.1–1.5], *p* = 0.003) significantly increased from day 0 to day 1 in patients with stunned myocardium but not in those without (P_bt_O_2_: *p* = 0.51, CI: *p* = 0.94, respectively).

### *Volume status and P*_*bt*_*O*_*2*_

Mean daily fluid input and fluid balance were 4.9 ± 1.9 L and 0.7 ± 1.7 L, respectively. Neither daily fluid input nor fluid balance was associated with absolute P_bt_O_2_ levels (*p* = 0.94, *p* = 0.85, respectively) or the occurrence of BTH (*p* = 0.68, *p* = 0.71, respectively). Low GEDI (< 680 mL/m^2^) or high SVV (> 10%) were observed in 31% and 25% of the monitored time, respectively; P_bt_O_2_ levels were similar in preload dependent patients as compared to episode of euvolemia (low GEDI: *p* = 0.53, high SVV: *p* = 0.30, respectively). A higher △CI was not associated with SVV > 10% (*p* = 0.065).

### *Fluid challenges and P*_*bt*_*O*_*2*_

**Fig. 5:**
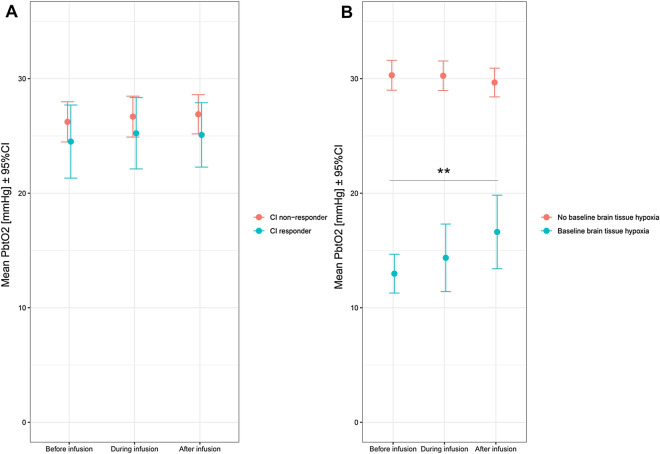
**A** P_bt_O_2_ did not change (*p* = 0.885) as a response to fluid boluses both in CI responders and non-responders (*p* = 0.92, *p* = 1.00). **B** P_bt_O_2_ increased as a response to fluid boluses in the setting of baseline brain tissue hypoxia (*p* = 0.002)

In total, 198 fluid boluses were analyzed in 48 patients, consisting of colloid (36%) or crystalloid fluid (64%) administration; 50 fluid boluses (25%) resulted in clinically significant CI increases (from 3.5 ± 1.2 to 4.4 ± 1.6 L/min/m^2^, *p* < 0.001). A significant CI increase was more frequently achieved with colloids than with crystalloids (38% vs. 18%, *p* = 0.004). P_bt_O_2_ did not change (*p* = 0.89) as a response to fluid boluses, both in fluid responders and non-responders (*p* = 0.92, *p* = 1.00, respectively; Fig. 5A, Additional file 1). An increase in P_bt_O_2_ was observed in the setting of BTH at baseline (*n* = 49/198, 25%; from 13 ± 6 to 16 ± 11 mmHg; OR = 13.3 [95% CI 2.6–67.4], *p* = 0.002; days 1–9: OR = 15.8 [95% CI 2.7–93.6], *p* = 0.002), even after adjusting for CI, MAP, ICP, PaO2, age, and H&H grade (adjusted OR = 190.4 [95% CI 2.8–12,810.5], *p* = 0.015, Fig. 5B). Consequently, during these episodes in 26% normal P_bt_O_2_ levels were achieved after the fluid challenge. Changes of selected variables in response to the fluid administration are given in Table 2 and Additional file 1.

**Table 2:** Changes of variables pre-, during, and post-fluid administration in a total of 198 infusions in 48 patients

Variable	Pre infusion	During infusion	Post infusion	*p*-value**
P_bt_O_2_, mmHg	26 ± 10	26 ± 11	26 ± 10	0.885
BTH, percent	49 (25)	50 (25)	41 (21)	0.241
CI, L/min/m^2^	3.9 ± 1.1	3.9 ± 1.1	4.0 ± 1.2	** < 0.001**
SVV, percent	11 ± 6	11 ± 6	9 ± 5	** < 0.001**
MAP, mmHg	104 ± 15	104 ± 17	106 ± 16	0.116
CPP^a^, mmHg				0.264
IBK	73 ± 9	71 ± 10	74 ± 10	
BRU	109 ± 26	108 ± 28	109 ± 29	
ICP, mmHg	12 ± 8	12 ± 9	12 ± 10	0.582
HR, bpm	86 ± 15	86 ± 15	86 ± 17	0.973
PRx^b^	0.10 ± 0.28	0.08 ± 0.27	0.08 ± 0.30	0.187
Impaired autoregulation (PRx ≥ 0.2)^b^	21 (42)	20 (36)	19 (34)	0.264

*P*_*bt*_*O*_*2*_ brain tissue oxygenation, *BTH* brain tissue hypoxia, *CI* continuous cardiac index, *SVV* stroke volume variation, *MAP* mean arterial pressure, *CPP* cerebral perfusion pressure, *ICP* intracerebral pressure, *HR* heart rate, *PRx* pressure reactivity index

^a^IBK: zeroed at the level of the Monroe foramen, BRU: zeroed at the heart level

Data are given in mean ± SD or counts (%)

^b^Subanalysis in 15 patients

^**^p-values indicate the difference between pre (1 h before) and post (1 h after) fluid administration

## Discussion

In this study we report an association between the cardiac performance status, as assessed with CI, and brain oxygenation. Moreover, we could demonstrate an increase in P_bt_O_2_ after fluid challenges only in the setting of baseline brain tissue hypoxia.

We found a positive association between the cardiac performance reflected by a higher CI and higher brain tissue oxygen levels, which is in accordance with previous studies [15, 16]. Imaging studies, including positron emission tomography scans and xenon computed tomography, demonstrated that increases in cardiac output resulted in improved CBF [24, 25]. Accordingly, we found that increases in CI (∆CI) were associated with increases in P_bt_O_2_ which is supported by a significant interaction between the presence of BTH and CI increase arguing against a spontaneous P_bt_O_2_ increase.

Patients with myocardial stunning had higher P_bt_O_2_ levels, which likely reflects a treatment effect with inotropic agents. This is further supported be the observation that P_bt_O_2_ increased in parallel to CI in the very early phase in patients with myocardial stunning but not in those without. The pathophysiology of myocardial stunning after SAH is multifactorial, including the activation of the hypothalamic–pituitary–adrenal (HPA) axis, catecholamine surge, and sympathetic and parasympathetic dysregulation [7]. Positive inotropic drugs, such as dobutamine, levosimendan, or milrinone, are used in the setting of severely impaired left ventricular function which can be monitored with the PiCCO (CI) [21]. Previous studies reported a drug-induced improvement of regional CBF (rCBF) in ischemic regions secondary to dobutamine administration in SAH patients underlining its positive effect on the cerebrovascular besides the cardiac system. P_bt_O_2_ may reflect an important physiologic target variable to monitor treatment efficacy. In turn, the presence of BTH should elicit adequate treatment interventions to prevent brain tissue hypoxia. It is important to keep in mind that we followed a P_bt_O_2_ targeted treatment approach in these patients aiming at the prevention of BTH [26, 27].

Although we could not replicate the results of Kurtz et al., where fluid challenges resulted in increases of P_bt_O_2_ levels, particularly in fluid responders (> 10% CI) [15], we found that P_bt_O_2_ rose reactive to fluid boluses administered during episodes of brain tissue hypoxia. Even more important, in 26% of these episodes an increase to normal P_bt_O_2_ levels was achieved, indicating a clinically relevant finding, especially as higher P_bt_O_2_ levels were associated with improved outcomes in our cohort. This finding is supported by a previous study where the administration of saline boluses resulted in increases of rCBF in areas with low flow at baseline and thus being most vulnerable to ischemia [24]. In the remaining fluid challenges, we could not find an increase in P_bt_O_2_ levels and during some episodes P_bt_O_2_ even decreased, despite following a goal-directed fluid management with the PiCCO device which allows the prediction of fluid responders. Hemodilution after fluid administration may have outweighed the improvement of the cardiac output in these patients [16]. We could even not demonstrate rising P_bt_O_2_ levels in fluid responders as reflected by CI increases. This is an important finding and might indicate that the brain is independent on systemic changes under certain conditions again supporting individualized management with the need of additional monitoring of brain physiologic data. One explanation may lie in the independence of P_bt_O_2_ regulation based on the complex interaction between P_bt_O_2_ and systemic parameters. P_bt_O_2_ has been shown to be a surrogate of CBF [16, 28] although it does not only reflect oxygen delivery but is the net sum of oxygen delivery, consumption, and diffusion [14]. Therefore, factors, such as impaired neurovascular coupling, cerebral edema, fever, spreading depolarizations, neuroinflammation, or lung injury, may have influenced our results [29–33]. Other variables that affect the relation between fluid management and CBF or P_bt_O_2_ include the vascular tone and permeability of vessels with third space losses in the presence of leaky capillaries and hypoalbuminemia, a condition commonly observed in critically ill and sedated patients [20, 34]. Also, the state of cerebral autoregulation potentially impacts on P_bt_O_2_ responses. Importantly, CBF and P_bt_O_2_ are expected to rise in parallel with CPP and CI increases in the presence of impaired autoregulation, while P_bt_O_2_ may be independent on CPP/CI changes across intact autoregulation ranges [22]. We found a robust association between cardiac output and brain oxygenation irrespective of the autoregulatory state in a subset of 18 patients. In a considerable number of episodes, the autoregulation was impaired during fluid challenges; however, we could not integrate the autoregulatory state in episodes of BTH at baseline due to restricted patient numbers (5 remaining patients). Impaired autoregulation may explain the missing interaction between CI and CPP when assessing the effect of CI on P_bt_O_2_ as one would expect a more pronounced increase in CBF/P_bt_O_2_ secondary to a CI increase when the CPP is low, while patients with an already high cerebral blood supply (with a high CPP) would less benefit from CI increase.

We did not find an association between the volemic status and P_bt_O_2_. Neither the absolute fluid input nor a preload dependent status influenced absolute P_bt_O_2_ levels. Volume expansion increases cardiac filling pressures with a subsequent increase of the cardiac output according to the Frank-Starling mechanism, which in turn could enhance cerebral blood flow and thereby increased oxygen delivery [35, 36]. Another mechanism theoretically resulting in improved microcirculation in ischemic areas is decreased blood viscosity achieved through volume administration [37]. However, these effects may be outweighed by complications arising with fluid overload, such as cardiac failure, or pulmonary and cerebral edema [3]. Even more important, hypervolemia as part of the triple-H concept did not result in improved CBF and better functional outcomes in clinical practice [16, 25, 38]. Hemodilution with reduced blood viscosity is associated with decreased hematocrit values which can contribute to reduced oxygen delivery to the brain [39]. In this regard it is important to mention that we aimed at euvolemia in all our patients which was mainly directed by the PiCCO system and clinical examination.

Some limitations to this study merit consideration. Firstly, only patients requiring continuous cardiac and neuromonitoring were included in this study limiting the transferability of our results to good-grade SAH patients. This is also reflected in the high percentage of poor functional outcomes three months after ictus. Secondly, due to the retrospective analysis of prospectively collected data, we do not know the exact indication of fluid challenges, which mainly include fluid maintenance and replacement. To minimize this limitation, we performed a separate analysis for crystalloid and colloid fluids and found that colloids more frequently resulted in CI and MAP increases as expected; however, there was no difference on P_bt_O_2_ responses. Thirdly, we cannot conclude whether only fluid challenges or simultaneous effects of other interventions resulted in P_bt_O_2_ rises because our institutional protocols foresee interventions without a hierarchical order to treat brain tissue hypoxia. Moreover, we focused on fluids only and did not check for CPP/MAP changes secondary to vasopressor adaptions as this was beyond the scope of the study. However, we give cumulative daily doses of the most important vasopressors used. Fourthly, we did not include other cardiac parameters as derived from echocardiography or blood results as they were not available in all patients included. Fifthly, SVV should only be integrated into clinical decision making if various conditions, including a full mechanical ventilation mode, and the absence of arrhythmias, are fulfilled. Moreover, higher tidal volumes or higher PEEP levels may produce higher variations, and changes in lung or chest compliance or patient position may affect readings. We only included SVV readings during a fully controlled ventilation mode (73%) but were unable to exclude episodes with arrhythmias. Based on our approach of retrospective analysis, we could not adequately define hypovolemia, where low GEDI and high SVV should be associated with a clinical problem, such as hypotension, oliguria, or increased heart rate without another explanation than hypovolemia. Lastly, we may have missed episodes of BTH by cropping the analysis at day 11. However, both P_bt_O_2_ and PiCCO monitoring were performed in the most vulnerable time only and including data points thereafter would have skewed results toward the most severely injured patients.

## Conclusions

This study indicates that cardiac performance and brain oxygenation are moderately linked in poor-grade SAH patients. Based on the findings that fluid challenges only resulted in improved P_bt_O_2_ levels when brain tissue hypoxia was evident at baseline, there is the need to use both, advanced cardiac and neuromonitoring, in poor-grade SAH patients in order to individualize critical care management.

## Supplementary Information

**Additional file 1**: **Figure S1**. Institutional protocol to treat brain tissue hypoxia in IBK. **Figure S2**. Institutional protocol to treat brain tissue hypoxia in BRU. **Table S1**. Baseline characteristics, complications, and outcomes stratified by the two study sites. **Table S2**. Association between CI and P_bt_O_2_ (GEE model). **Table S3**. Changes of variables pre, during, and post fluid administration in a total of 198 infusions stratified by brain tissue hypoxia vs. normal brain tissue oxygenation at baseline. **Table S4**. Changes of variables pre, during, and post fluid administration in a total of 198 infusions stratified by CI responders and non-responders. **Table S5**. Changes of variables pre, during, and post fluid administration in a total of 198 infusions stratified by crystalloids vs colloids.

## Data Availability

The data that support the findings of this study are available from the corresponding author, upon reasonable request.

## Abbreviations

SAH: Subarachnoid hemorrhage
PiCCO: Pulse contour cardiac output
CI: Cardiac index
GEDI: Global end-diastolic index
SVV: Stroke volume variation
P_bt_O_2_: Brain tissue oxygenation
BTH: Brain tissue hypoxia
CBF: Cerebral blood flow
CT: Computed tomography
ICP: Intracranial pressure
HR: Heart rate
CPP: Cerebral perfusion pressure
MAP: Mean arterial pressure
DCI: Delayed cerebral ischemia
H&H: Hunt&Hess
PRx: Pressure reactivity index
