# Supplementary material for: The effect of the volemic and cardiac status on brain oxygenation in patients with subarachnoid hemorrhage: a bi-center cohort study
Source: Ann Intensive Care. 2021 Dec 16;11:176. doi: 10.1186/s13613-021-00960-z (PMC8677880; doi:10.1186/s13613-021-00960-z)
Supplement: Supplementary file 1 — Additional file 1: Figure S1. Institutional protocol to treat brain tissue hypoxia in IBK. Figure S2. Institutional protocol to treat brain tissue hypoxia in BRU. Table S1. Baseline characteristics, complications, and outcomes stratified by the two study sites. Table S2. Association between CI and PbtO2 (GEE model). Table S3. Changes of variables pre, during, and post fluid administration in a total of 198 infusions stratified by brain tissue hypoxia vs. normal brain tissue oxygenation at baseline. Table S4. Changes of variables pre, during, and post fluid administration in a total of 198 infusions stratified by CI responders and non-responders. Table S5. Changes of variables pre, during, and post fluid administration in a total of 198 infusions stratified by crystalloids vs colloids. [file 13613_2021_960_MOESM1_ESM.docx]

**Additional File 1**

| **Supplemental Table 1. Baseline characteristics, complications, and outcomes stratified by the two study sites** | | | |
| --- | --- | --- | --- |
| **Clinical Characteristics and Premedical History** | **IBK**  **N=39** | **BRU**  **N=21** | **p-value*** |
| Age in years | 58 (48-66) | 52 (46-64) | 0.876 |
| Female sex | 28 (72) | 9 (43) | 0.050 |
| Chronic heart failure | 0 (0) | 0 (0) | - |
| Previous myocardial infarction | 1 (3) | 2 (10) | 0.278 |
| Pre-existing hypertension | 14 (36) | 8 (38) | 1.000 |
| Smoking history | 17 (44) | 7 (33) | 0.582 |
| Admission H&H grade | 5 (3-5) | 3 (2-5) | 0.073 |
| GCS at ICU admission | 3 (3-8) | 7 (3-13) | 0.059 |
| LOC at ictus | 22 (56) | 14 (67) | 0.582 |
| **Admission Radiological Characteristics** | | |  |
| Modified Fisher Scale | 4 (3-4) | 4 (3-4) | 0.867 |
| ICH present on admission CT-scan | 17 (44) | 13 (62) | 0.279 |
| Hydrocephalus requiring EVD placement | 34 (87) | 8 (38) | **<0.001** |
| Global cerebral edema | 14 (36) | 10 (48) | 0.418 |
| **Aneurysm Treatment** | | |  |
| Endovascular coiling | 16 (41) | 15 (71) | 0.053 |
| Neurosurgical clipping | 22 (56) | 5 (24) |  |
| Non-aneurysmal SAH | 1 (3) | 1 (5) |  |
| **Complications and Treatments** |  |  |  |
| Neurogenic myocardial stunning | 15 (39) | 8 (38) | 1.00 |
| Pneumonia | 28 (72) | 14 (67) | 0.771 |
| Ventriculitis | 6 (15) | 1 (5) | 0.404 |
| Vasospasm | 34 (87) | 14 (67) | 0.090 |
| Delayed cerebral ischemia | 14 (36) | 11 (52) | 0.276 |
| Noradrenaline, daily dose in mg** | 9 (3-15) | 41 (11-93) | **<0.001** |
| Dobutamine, daily dose in mg** | 155 (0-322) | 0 (0-14) | 0.461 |
| Phenylephrine, daily dose in mg** | 0 (0-37) | 0 (0-0) | **<0.001** |
| P_bt_O_2_ in mmHg** | 26±11 | 26±9 | 0.611 |
| BTH, percent of monitoring time** | 24 | 21 | 0.786 |
| **Outcome Characteristics** | | |  |
| Length of ICU stay in days | 30 (24-44) | 19 (11-30) | **0.002** |
| In-hospital mortality | 5 (13) | 9 (43) | **0.012** |
| 3-month mRS | 4 (2-5) | 5 (3-6) | 0.080 |
| SAH - subarachnoid hemorrhage, H&H - Hunt and Hess, LOC - loss of consciousness, ICH - intracerebral hemorrhage, EVD - external ventricular drain, BTH – brain tissue hypoxia, mRS - modified Rankin Scale. Data are given in mean±SD, median (IQR) and counts (%).  *Differences across patients from the two sites were assessed with the T-test, Mann-Whitney U test, Qui square test, Fishers exact test, and GEE models in repeated measurements.  ** within the first 10 days | | | |

| **Supplemental Table 2. Association between CI and P_bt_O_2_ (GEE model)** | | |
| --- | --- | --- |
| **Variable** | **Wald** | **p-value*** |
| CI | 6.8 | 0.009 |
| MAP | 3.5 | 0.063 |
| ICP | 0.5 | 0.474 |
| Age | 3.4 | 0.067 |
| H&H | 0.4 | 0.550 |
| PaO2 | 8.3 | 0.004 |
| CI*CPP (interaction) | 2.7 | 0.098 |
| Dependent variable: P_bt_O_2_  P_bt_O_2_ – brain tissue oxygenation, CI – continuous cardiac index, MAP – mean arterial pressure, CPP – cerebral perfusion pressure, ICP – intracerebral pressure, PaO2 - partial pressure of oxygen, H&H - Hunt and Hess | | |

| **Supplemental Table 3. Changes of variables pre- during and post fluid administration in a total of 198 infusions stratified by brain tissue hypoxia vs. normal brain oxygenation at baseline** | | | | | | | | |
| --- | --- | --- | --- | --- | --- | --- | --- | --- |
|  | Brain tissue hypoxia at baseline, n=49 | | | | Normal brain oxygenation at baseline, n=149 | | | |
| **Variable** | **Pre infusion** | **During infusion** | **Post infusion** | **p-value**** | **Pre infusion** | **During infusion** | **Post infusion** | **p-value**** |
| P_bt_O_2_, mmHg | 13±6 | 14±10 | 16±11 | **0.002** | 30±7 | 30±8 | 30±8 | 0.154 |
| BTH, percent | 49 (100) | 43 (88) | 32 (64) | **<0.001** | 0 (0) | 7 (5) | 11 (7) | **<0.001***** |
| CI, L/min/m^2^ | 3.6±1.0 | 3.7±1.0 | 3.7±1.0 | **0.036** | 4.0±1.1 | 4.0±1.2 | 4.1±1.2 | **0.004** |
| SVV, percent | 9±5 | 10±5 | 8±4 | **0.002** | 12±6 | 12±6 | 9±5 | **<0.001** |
| MAP, mmHg | 106±20 | 105±21 | 107±21 | 0.164 | 104±14 | 104±16 | 105±14 | 0.283 |
| CPP*, mmHg |  |  |  | 0.785 |  |  |  | 0.059 |
| IBK | 73±10 | 70±11 | 73±12 |  | 73±9 | 72±9 | 74±10 |  |
| BRU | 105±35 | 105±37 | 101±42 |  | 112±18 | 111±20 | 114±17 |  |
| ICP, mmHg | 15±13 | 15±13 | 16±16 | 0.229 | 11±6 | 11±6 | 11±6 | 0.202 |
| HR, bpm | 82±16 | 84±16 | 84±22 | 0.832 | 87±14 | 87±15 | 87±15 | 0.625 |
| P_bt_O_2_ – brain tissue oxygenation, BTH – brain tissue hypoxia; CI – continuous cardiac index, SVV- stroke volume variation, MAP – mean arterial pressure, CPP – cerebral perfusion pressure, ICP – intracerebral pressure, HR – heart rate.  *IBK: zeroed at the level of the Monroe foramen, BRU: zeroed at the heart level  Data are given in mean±SD or counts (%).  ** P-values indicate the difference between pre (one hour before) and post (one hour after) fluid administration.  *** Mean P_bt_O_2_ levels decreased from 22±4 mmHg before fluid administration to 18±2 mmHg post fluid administration. | | | | | | | | |

| **Supplemental Table 4. Changes of variables pre- during and post fluid administration in a total of 198 infusions stratified by CI responders and non-responders.** | | | | | | | | |
| --- | --- | --- | --- | --- | --- | --- | --- | --- |
|  | Cardiac responders (∆CI ≥10%), n=50 | | | | Cardiac non-responders (∆CI <10%), n=148 | | | |
| **Variable** | **Pre infusion** | **During infusion** | **Post infusion** | **p-value**** | **Pre infusion** | **During infusion** | **Post infusion** | **p-value**** |
| P_bt_O_2_, mmHg | 25±11 | 25±11 | 25±10 | 0.917 | 26±10 | 27±11 | 27±11 | 0.999 |
| BTH, percent | 14 (29) | 14 (28) | 12 (24) | 0.513 | 34 (25) | 36 (24) | 29 (20) | 0.432 |
| CI, L/min/m^2^ | 3.5±1.2 | 3.9±1.5 | 4.4±1.6 | **<0.001** | 4.0±1.2 | 3.9±1.0 | 3.9±1.0 | 0.126 |
| SVV, percent | 12±7 | 11±7 | 9±6 | **0.001** | 11±5 | 11±5 | 9±5 | **<0.001** |
| MAP, mmHg | 103±16 | 105±19 | 107±17 | 0.125 | 105±16 | 104±16 | 105±16 | 0.788 |
| CPP*, mmHg |  |  |  | **<0.001** |  |  |  | 0.447 |
| IBK | 70±8 | 72±10 | 76±11 |  | 74±9 | 72±10 | 73±10 |  |
| BRU | 101±24 | 97±30 | 99±39 |  | 112±26 | 112±27 | 112±26 |  |
| ICP, mmHg | 14±9 | 13±12 | 13±12 | 0.589 | 12±8 | 12±7 | 12±9 | 0.460 |
| HR, bpm | 82±13 | 83±13 | 84±13 | **0.014** | 87±15 | 87±16 | 87±18 | 0.579 |
| P_bt_O_2_ – brain tissue oxygenation, BTH – brain tissue hypoxia; CI – continuous cardiac index, SVV- stroke volume variation, MAP – mean arterial pressure, CPP – cerebral perfusion pressure, ICP – intracerebral pressure, HR – heart rate.  *IBK: zeroed at the level of the Monroe foramen, BRU: zeroed at the heart level  Data are given in mean±SD or counts (%).  ** P-values indicate the difference between pre (one hour before) and post (one hour after) fluid administration. | | | | | | | | |

| **Supplemental Table 5. Changes of variables pre- during and post fluid administration in a total of 198 infusions stratified by crystalloids vs colloids.** | | | | | | | | |
| --- | --- | --- | --- | --- | --- | --- | --- | --- |
|  | Colloids, n=72 | | | | Crystalloids, n=126 | | | |
| **Variable** | **Pre infusion** | **During infusion** | **Post infusion** | **p-value**** | **Pre infusion** | **During infusion** | **Post infusion** | **p-value**** |
| P_bt_O_2_, mmHg | 27±10 | 28±10 | 28±11 | 0.107 | 25±11 | 25±11 | 25±10 | 0.468 |
| BTH, percent | 14 (20) | 16 (22) | 13 (18) | 0.530 | 34 (30) | 34 (27) | 28 (22) | 0.215 |
| CI, L/min/m^2^ | 3.8±1.2 | 3.8±1.1 | 4.1±1.4 | **<0.001** | 3.9±1.0 | 4.0±1.2 | 4.0±1.1 | 0.053 |
| SVV, percent | 12±7 | 11±6 | 8±5 | **<0.001** | 11±6 | 11±6 | 10±5 | **<0.001** |
| MAP, mmHg | 100±12 | 99±14 | 103±14 | **0.031** | 107±17 | 107±18 | 107±17 | 0.884 |
| CPP*, mmHg |  |  |  | **0.012** |  |  |  | 0.941 |
| IBK | 73±9 | 70±9 | 76±10 |  | 73±9 | 73±10 | 74±11 |  |
| BRU | - | - | - |  | 109±26 | 108±28 | 109±29 |  |
| ICP, mmHg | 11±5 | 11±5 | 11±5 | 0.940 | 13±10 | 13±10 | 13±11 | 0.778 |
| HR, bpm | 84±14 | 84±14 | 83±14 | 0.213 | 87±15 | 87±15 | 88±18 | 0.471 |
| P_bt_O_2_ – brain tissue oxygenation, BTH – brain tissue hypoxia; CI – continuous cardiac index, SVV- stroke volume variation, MAP – mean arterial pressure, CPP – cerebral perfusion pressure, ICP – intracerebral pressure, HR – heart rate.  *IBK: zeroed at the level of the Monroe foramen, BRU: zeroed at the heart level  Data are given in mean±SD or counts (%).  ** P-values indicate the difference between pre (one hour before) and post (one hour after) fluid administration. | | | | | | | | |

Supplemental Figure 1. Institutional protocol to treat brain tissue hypoxia in IBK.

**BRAIN TISSUE HYPOXIA (PbtO2 <20mmHg)**


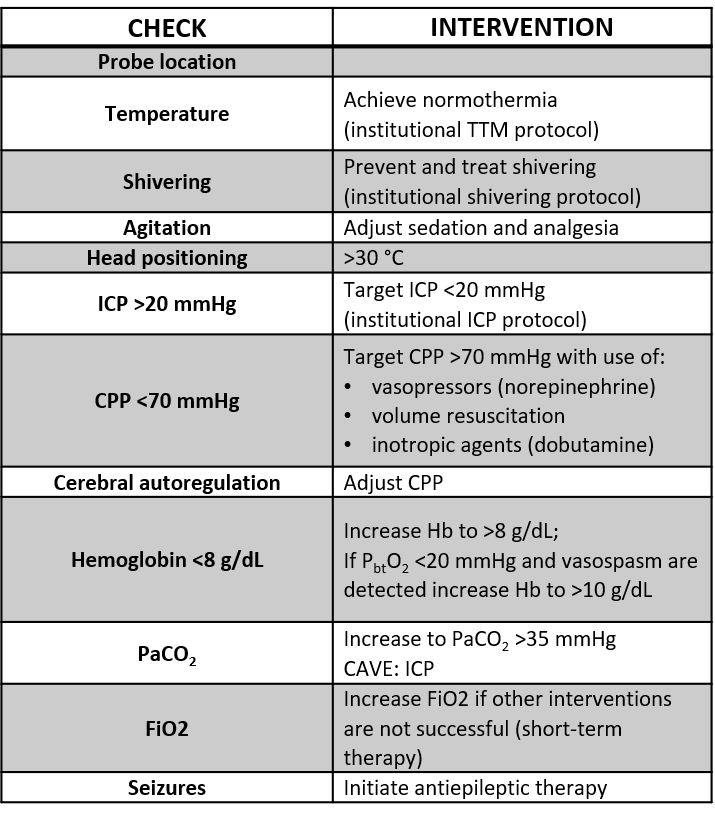


Supplemental Figure 2. Institutional protocol to treat brain tissue hypoxia in BRU.


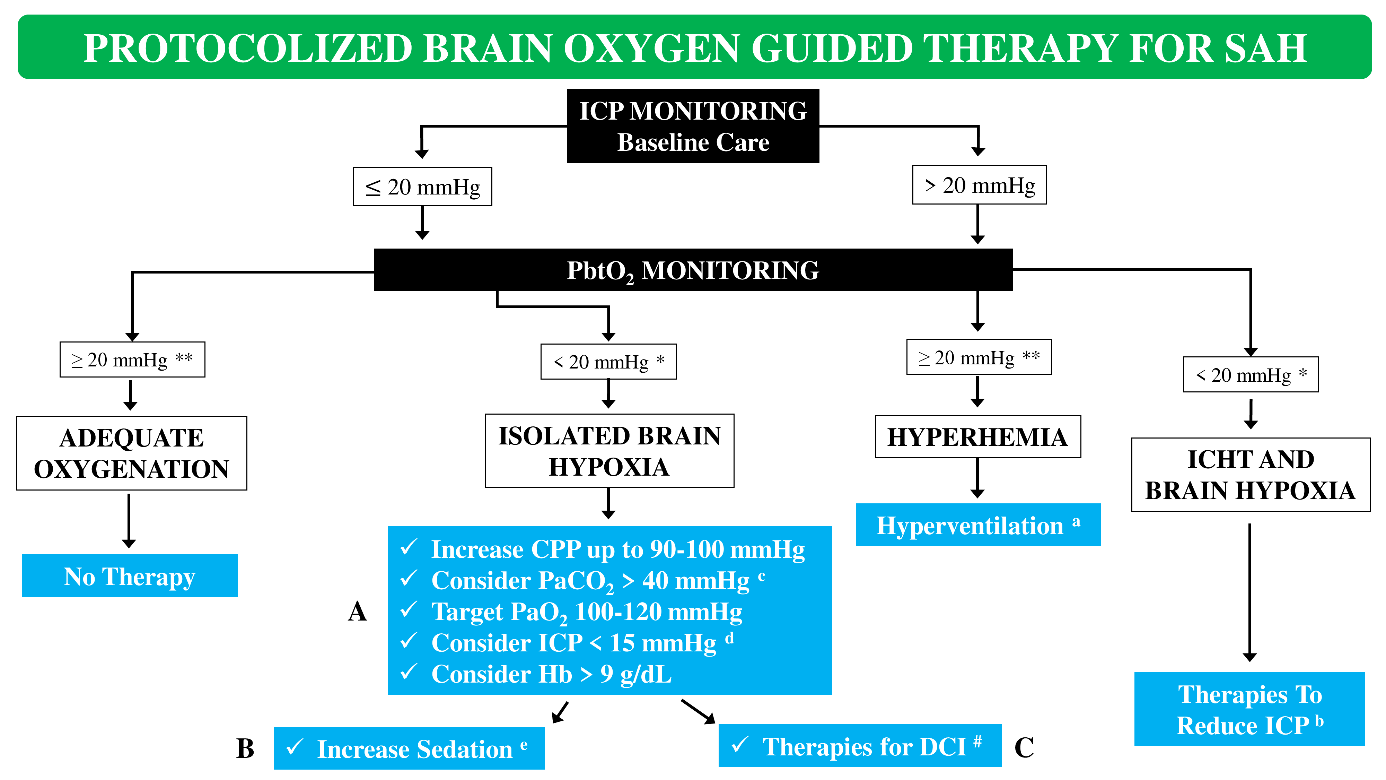


The order of which intervention to be started first is dependent on patients’ condition and treating physician

A = Suspected low oxygen delivery

a if ICP is between 20-25 mmHg and patient is awakening, no specific therapy could be considered.

c if ICP remains below 20 mmHg

d if ICP is between 15 and 20 mmHg with tissue hypoxia

B = Suspected increased oxygen consumption (i.e. agitation; seizures; inadequate sedation on EEG monitoring)

C = Suspected delayed cerebral ischemia (DCI): diagnosis of DCI is based on a combination of parameters (i.e. clinical deterioration when clinical examination is available or EEG monitoring, PbtO2 and trans-cranial Doppler for unconscious patients) and the demonstration of altered cerebral perfusion on CT-perfusion $\pm$narrowing of intracranial vessels (angiography or angio-CT scan).

#These includes: a) induced hypertension; b) intra-arterial vasodilators; c) cerebral angioplasty; d) rescue therapies (i.e. intra-carotid catheter for continuous infusion of vasodilators; therapeutic hypothermia; steroids; high-dose magnesium)
